# Stress during pregnancy alters temporal and spatial dynamics of the maternal and offspring microbiome in a sex-specific manner

**DOI:** 10.1038/srep44182

**Published:** 2017-03-07

**Authors:** Eldin Jašarević, Christopher D. Howard, Ana M. Misic, Daniel P. Beiting, Tracy L. Bale

**Affiliations:** 1 Department of Biomedical Sciences, School of Veterinary Medicine, University of Pennsylvania, Philadelphia, PA 19104, USA; 2 Center for Host-Microbial Interactions, School of Veterinary Medicine, University of Pennsylvania, Philadelphia, PA 19104, USA; 3 Department of Pathobiology, School of Veterinary Medicine, University of Pennsylvania, Philadelphia, PA 19104, USA.

## Abstract

The microbiome is a regulator of host immunity, metabolism, neurodevelopment, and behavior. During early life, bacterial communities within maternal gut and vaginal compartments can have an impact on directing these processes. Maternal stress experience during pregnancy may impact offspring development by altering the temporal and spatial dynamics of the maternal microbiome during pregnancy. To examine the hypothesis that maternal stress disrupts gut and vaginal microbial dynamics during critical prenatal and postnatal windows, we used high-resolution 16S rRNA marker gene sequencing to examine outcomes in our mouse model of early prenatal stress. Consistent with predictions, maternal fecal communities shift across pregnancy, a process that is disrupted by stress. Vaginal bacterial community structure and composition exhibit lasting disruption following stress exposure. Comparison of maternal and offspring microbiota revealed that similarities in bacterial community composition was predicted by a complex interaction between maternal body niche and offspring age and sex. Importantly, early prenatal stress influenced offspring bacterial community assembly in a temporal and sex-specific manner. Taken together, our results demonstrate that early prenatal stress may influence offspring development through converging modifications to gut microbial composition during pregnancy and transmission of dysbiotic vaginal microbiome at birth.

Early environmental perturbations, such as chronic stress or infection during pregnancy, are associated with profound disruption to the developing central nervous system, including circuits that regulate stress, affect, and cognition^1^,^2^,^3^,^4^,^5^. Rodent and primate models of early life adversity demonstrate alterations to the gut-brain axis that mimic endophenotypes of neurodevelopmental disorders, including increased intestinal permeability, visceral hypersensitivity, stress pathway dysregulation, altered development of the enteric nervous system, and changes to behavior^6^,^7^,^8^,^9^,^10^,^11^,^12^,^13^,^14^,^15^,^16^,^17^,^18^,^19^. The mechanisms through which fetal antecedents contribute to gut-brain dysfunction likely involve dynamic interactions between the maternal and fetal compartments^1^. One potential interaction that has yet to be explored is the prolonged effect of stress on bacterial communities during pregnancy.

During pregnancy a continuous maternal supply of nutrients and substrates to the fetus is essential to support normal growth and development^20^. This nutritional and metabolic demand of the developing fetus is likely modulated by the capacity of the maternal gut microbiome to harvest and produce these substrates^21^,^22^. Indeed, metabolites of maternal gut microbial origin, such as short chain fatty acids, saccharides, and amino acids cross the placental barrier, enter fetal circulation, and contribute to the formation of the blood-brain barrier and innate immune development^23^,^24^. Previous work in our laboratory has shown that maternal stress experience during the first week of pregnancy results in alterations to placental transfer of nutrients in a sex-specific manner^25^,^26^,^27^. As maternal nutritional status influences placental function, alterations to the structure and metabolic potential of the maternal bacterial microbiome may represent a currently uncharacterized association through which maternal stress experience impacts offspring outcomes^28^,^29^.

During parturition the bacterial communities within the maternal vagina also play a significant role in offspring postnatal development^6^,^7^,^8^,^9^,^13^,^14^,^15^,^18^,^30^,^31^. As maternal vaginal bacterial communities provide the primary inoculum that colonize the neonate gut at birth, the composition of these pioneer communities contributes disproportionally to long-term health outcomes^32^,^33^,^34^,^35^,^36^. Maternal factors, such as maternal immune activation, diet, antibiotics, and mode of delivery impact vertical transmission of vaginal communities^33^,^34^,^36^,^37^,^38^,^39^,^40^. As a result, transmission of dysbiotic vaginal bacterial communities has been proposed to disrupt neonate-microbe interactions necessary for immune education, metabolism, and neurodevelopment^37^,^41^,^42^,^43^,^44^. Consistent with this hypothesis, previous work in our laboratory has shown that early prenatal stress decreased the relative abundance of the commensal *Lactobacillus* in the maternal vagina and neonate gut at colonization. Further, these paralleled changes to offspring gut and plasma metabolite profiles related to impaired energy metabolism and availability and sex- and brain region-specific changes in amino acid transport^18^. Transplantation of maternal vaginal microbiota to cesarean delivered mice was sufficient in recapitulating bacterial load observed in vaginally delivered offspring, recapitulating recent evidence in infants^18^,^34^. While these results show that maternal stress experience impacts microbial community composition and elicits metabolic consequences at birth, the lasting consequences of transmission of dysbiotic vaginal communities on the assembly of offspring microbial communities remains unclear.

Therefore, to examine the hypothesis that maternal stress experience disrupts gut and vaginal microbial dynamics during critical prenatal and postnatal windows, we used a high-resolution sampling strategy coupled with 16S rRNA marker gene sequencing to examine outcomes in our mouse model of early prenatal stress, in which male, but not female, offspring demonstrate significant neurodevelopmental changes in hypothalamic and limbic circuits and in the regulation of stress responsivity, cognitive dysfunction, and post-pubertal growth^18^,^25^,^26^,^45^,^46^,^47^,^48^. This approach provides an opportunity to draw novel associations between the effects of stress during pregnancy on the structure and functional potential of maternal bacterial communities and sex-specific phenotypes that emerge in offspring as adults. Further, unique assemblages of microbiota that differentiate treatment groups as a function of time, sex, and their interaction were identified using a supervised machine learning technique. Together, these studies provide an in-depth timeline reconstruction of temporal and spatial kinetics of maternal microbial communities following stress exposure and subsequent influence on the assembly of offspring microbial communities.

## Results

### Stress alters maternal fecal microbial diversity, community, and composition during pregnancy

**Figure 1:**
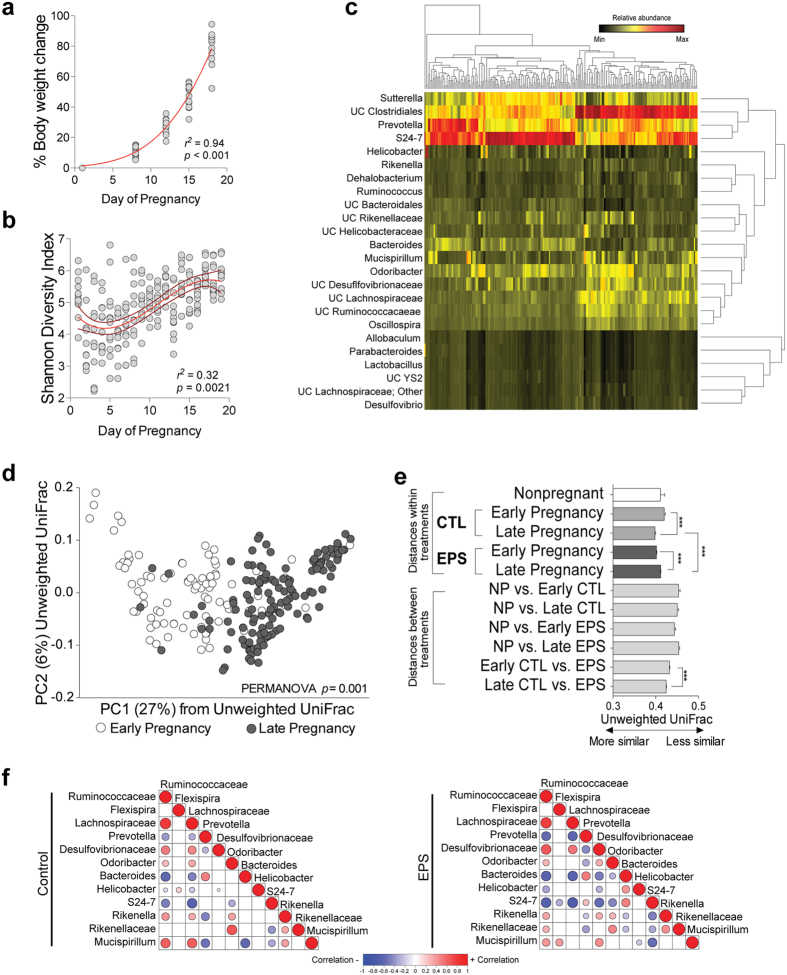
Stress alters pregnancy-associated microbial diversity, structure, and composition of fecal microbial communities. (**a**) Percent increase in total body weight across pregnancy demonstrates a slow and exponential growth phase during pregnancy in mice. There was no effect of stress on total body weight across pregnancy. N = 4–8 females per time point. (**b**) Shannon diversity index plotted against day of pregnancy, revealing that dynamic effects of pregnancy on community diversity. N = 4–8 females sampled daily per treatment, yielding 228 unique fecal samples in total; Non-parametric *t*-Test *p < 0.05, **p < 0.01, ***p < 0.001. (**c**) Heatmap depicting unsupervised clustering of samples (columns) of 97%-identity OTUs that exhibit at least 1% abundance in at least 2 females, demonstrating that taxa form two distinct clusters. The taxonomic annotation of each OTU is indicated to the left of rows. (**d**) Distinct structure of fecal bacterial communities during murine pregnancy. Communities clustered using PCoA of the unweighted UniFrac distance matrix, and each point corresponds to a sample that was collected daily during pregnancy. The percentage of variation explained by the PC is indicated on the axes, and colors correspond to samples collected either during early (white) and late (grey) pregnancy. N = 4–8 females sampled daily per treatment; PERMANOVA p-values based on 999 Monte Carlo simulations; PC, principal coordinate; PCoA, principal coordinates analysis.(**e**) Stress alterations to microbial community structure are limited to period of stress exposure. Average (±sem) unweighted UniFrac distances comparing phylogenetic configurations of fecal microbial communities of nonpregnant females, and control and stress-exposed females during early and late pregnancy. Mann Whitney U, ***p < 0.001. (**f**) Correlation matrix analysis of Random Forests-identified taxa that discriminatory across stage of pregnancy, demonstrating that stress disrupts co-occurrence and co-exclusion relationships among taxa. Empty boxes indicate nonsignificant correlations, while filled boxes are indicative of a significant correlation with a *P*-value ≤ 0.05 by Kendall’s test. The shading intensity of the bubble, along with size, is indicative of the Kendall rank correlation coefficient between matrices. Blue designates a negative correlation while red designates a positive correlation. N = 4–8 females sampled daily per treatment, yielding 228 unique fecal samples in total.

Mammalian pregnancy exhibits two distinct metabolic states: an anabolic stage characterized by a linear phase in maternal and fetal growth and a catabolic stage characterized by ketogenesis, gluconeogenesis, and accelerated placental transfer of nutrients, all of which support the exponential growth rate of the developing fetus^20^. In mice the linear growth phase is restricted to the first week of pregnancy, followed by an exponential growth phase the remaining two weeks of pregnancy (*r*^2^ = 0.94, *p* < 0.01, Fig. 1a). Previous work has shown that the maternal gut microbiome undergoes dynamic remodeling during pregnancy, favoring taxa with an increased capacity for extracting nutrients necessary to meet the nutritional and metabolic demand of pregnancy and in preparation for lactation^21^. To determine the impact of stress during the first week of pregnancy on murine gut microbial communities, fecal samples were obtained starting one day prior to mating and then daily during the course of pregnancy (nineteen days in mice) (Supplementary Fig. 1A). To determine temporal patterns in the bacterial community structure during pregnancy, alpha and beta diversity measures were calculated and compared as a function of day of pregnancy. Linear regression modeling revealed that community diversity across pregnancy was best predicted by a sigmoid function, characterized by an initial decrease during the first eight days and followed by an increase and stabilization during the remaining eleven days of pregnancy (Fig. 1b). Further, maternal body weight was significantly correlated Shannon diversity (*r*^2^ = 0.11, *p* = 0.011, Supplementary Fig. 1B). To determine the temporal patterning of bacterial taxa across pregnancy, unsupervised hierarchical clustering of 97%-identity OTUs was conducted. Consistent with Shannon diversity results, taxa clustered into two distinct stages (Fig. 1c, Supplemental Table 1).

As the Shannon Diversity Index and unsupervised clustering of 97%-identity OTUs indicated two distinct phases of pregnancy-associated community structure, the longitudinal data was binned into early (e.g., day 1–8) and late (e.g., day 9–19) stages of pregnancy and the bacterial community structures were assessed. Unweighted UniFrac distance analysis confirmed differences in community structure between these two periods (PERMANOVA, pseudo-F = 35.55, *p* = 0.001) (Fig. 1d). Inter-individual variation was significantly greater during early pregnancy than late pregnancy in control females, while stress decreased UniFrac distances among exposed females during both stages of pregnancy (Fig. 1e). Similarly, the community structure between control and stress-exposed females was significantly different during the early and late period of pregnancy (Fig. 1e).

To identify the bacterial signature that accounts for the observed shift in community structure following stress exposure, we applied Random Forests models to our longitudinal data as previously described^49^. This approach identified 13 discriminatory taxa whose relative abundance was differentially represented between early and late pregnancy in control females (Supplemental Fig. 2A). Members of the order Clostridiales of the phylum Firmicutes (*Lachnospiraceae* and *Ruminococcaceae*) increase during late pregnancy, and is paralleled by decreases in taxa within the Bacteroidetes phyla (*Prevotella* and *S24–7*) (Supplemental Fig. 2B). Reconfiguration of taxa within the Bacteroidetes phylum was observed in stress-exposed females, including enrichment of *Rikenellaceae* and *Odoribacter* with a concomitant loss of *Bacteroides* that emerged early in pregnancy and remained stable until parturition. Enrichment of the Desulfovibrionaceae family within the Proteobacteria phylum and *Mucispirillum* within the Deferribacteres phylum was observed during late pregnancy. Stress increased *Mucispirillum* abundance during the early stage of pregnancy and remained elevated throughout pregnancy (Supplemental Fig. 2B). As taxa exhibit significant co-occurrence and co-exclusion relationships, we determined whether stress exposure during pregnancy impacted these interactions by generating correlation matrices of Random Forests-identified taxa^50^. Significant correlations were detected between taxa across pregnancy that were disrupted by stress. For instance, the significant positive correlation between *Mucispirillum* and Lachnospiraceae in control dams disappeared in dams exposed to stress during pregnancy, while new correlations emerged in stress-exposed females, including a significant positive correlation between *Mucispirillum* and *Flexispira* (Fig. 1g). Taken together, these results suggest that stress during the first week of pregnancy altered community dynamics of maternal fecal bacteria.

Given the well-established functional redundancy of bacterial communities, shifts in community composition may not always parallel functional alterations^51^. To determine whether stress influenced the functional potential of bacterial communities, 16S rRNA gene sequencing data were used to predict the abundance of KEGG functional orthologs using phylogenetic investigation of communities by reconstruction of unobserved states (PICRUSt), which were then filtered, and discriminatory features were identified with Random Forests regressions^52^. Random Forests models revealed 30 functional categories that discriminate between early and late pregnancy in control females, including pathways involved in gluconeogenesis, lipid biosynthesis, amino acids and fatty acid metabolism, and N-glycan degradation. (Supplemental Fig. 2A,B). Together, these results show that maternal stress experience during the first week of pregnancy exerts lasting disruption to community structure, composition, and functional capacity.

### Chronic stress experience alters maternal vaginal bacteria composition

**Figure 2:**
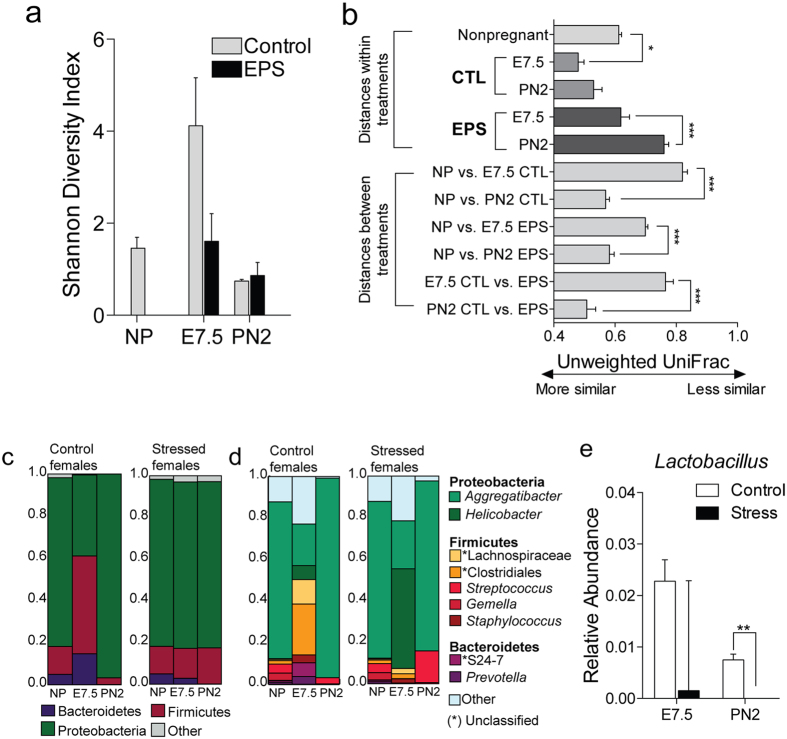
Stress alters diversity, structure, and composition of vaginal microbial communities. (**a**) Shannon diversity index plotted against sampling time point demonstrating that stress prevents the increase in microbial diversity at E7.5 as observed in control females. N = 5–8/day/treatment; Mann Whitney U, *p < *0.05*. (**b**) Stress alters microbial community structure in the vagina. Average (±sem) unweighted UniFrac distances comparing phylogenetic configurations of vaginal microbial communities of nonpregnant females, and control and stress-exposed females during early pregnancy and postpartum. N = 5–8/day/treatment; Mann Whitney U, *p < 0.05, ***p < 0.001. (**c**,**d**) Distribution of dominant taxa in control and stress-exposed females reveals an impact of stress on vaginal microbial community composition. (**c**) Left panel, mean phyla-level relative abundance of nonpregnant, pregnant, and postpartum vaginal samples. (**d**) Right panel, mean relative abundance of the most abundant taxa in vaginal communities sampled from nonpregnant, pregnant (E7.5), and postpartum (PN2) females. E7.5, gestation day 7.5; PN2, postnatal day 2; N = 5–8/day/treatment. (**e**) Vaginal *Lactobacillus* relative abundance is significantly decreased at PN2 in females exposed to stress compared with control females PN2, postnatal day 2; N = 5–8/day/treatment. Mann Whitney U, **p < 0.01.

As the maternal vaginal bacteria provide the primary inoculum of bacterial communities that colonize the neonate gut at birth, we next assessed the impact of stress on these communities. To determine whether temporal patterns of the maternal vaginal bacteria are impacted by stress during pregnancy and the postpartum period, vaginal fluid samples were obtained a day prior to mating, at the end of the first week of pregnancy (i.e., end of stress exposure), and 48 h postpartum. A significant increase in the vaginal Shannon Diversity Index was observed on gestational day 7.5 (E7.5) in control females relative to nonpregnant and postpartum control females (*p*s < 0.05, *t* test), while markedly blunted the effect of early pregnancy on vaginal community structure (Fig. 2a). Similarly, a difference in UniFrac distances emerged between nonpregnant and the E7.5 vaginal communities of control females. The vaginal community structure was also significantly different between control and stress-exposed females (*p*s < 0.05, permutation test) (Fig. 2b).

Given the stress alterations to community structure, we next examined whether these alterations were lasting or transient and therefore inconsequential to offspring reprogramming. The phylum Proteobacteria dominated the nonpregnant and postpartum murine vagina regardless of treatment (Fig. 2c) and stress-exposed females failed to exhibit a bloom of the Firmicutes and Bacteroidetes phyla at E7.5 compared with control females (Fig. 2c). At the genus level, members of *Aggregatibacter* constituted the dominant vaginal taxa in nonpregnant and postpartum murine vaginas (Fig. 2c). The Firmicutes and Bacteroidetes bloom in control females at E7.5 was driven by an expansion of members of unclassified *Lachnospiraceae, Clostridiales*, S24-7, and *Prevotella* (Fig. 2c). Consistent with alpha diversity measures, stress-exposed females showed a further expansion of Proteobacteria at E7.5, predominantly by members of the *Helicobacter* genus (Fig. 2c). In addition, relative abundance of vaginal *Lactobacillus* was decreased at PN2 in females exposed stress (*p* = 0.0044, Mann Whitney U, Fig. 2d). These results show that stress during pregnancy affects bacterial community structure and composition within the vagina, suggesting the possibility that these dysbiotic communities are transmitted to offspring during birth.

### Shared overlap between maternal and offspring communities is determined by offspring age

**Figure 3:**
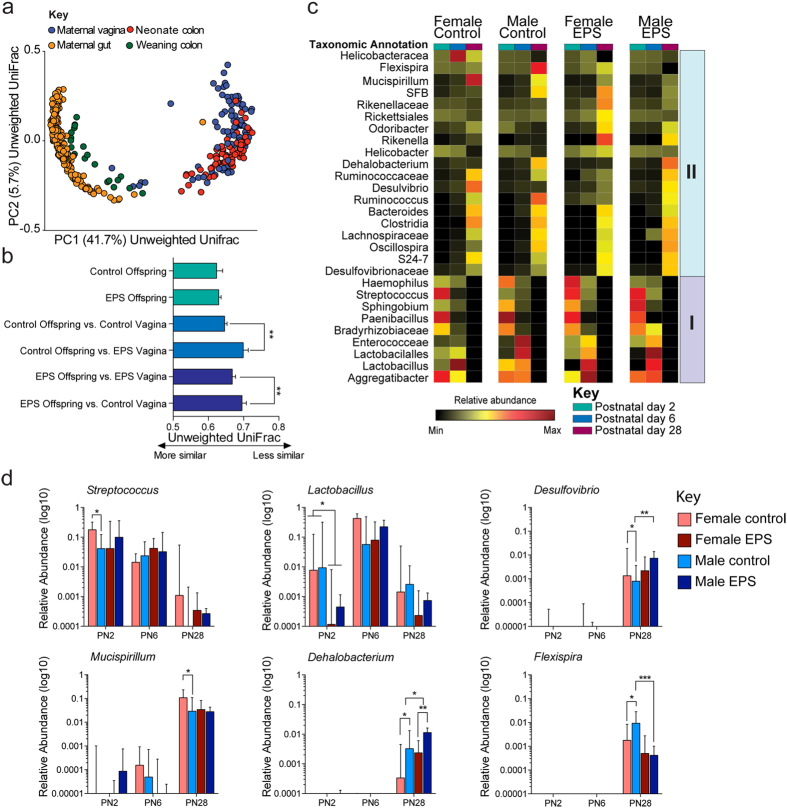
Effects of EPS on bacterial communities are sex-specific in offspring. (**a**) Newborn microbiota profiles overlap maternal vaginal microbial profiles while the weaning microbiota are similar to maternal gut microbial communities, providing an important proof-of-concept that colonization and subsequent assembly of the murine microbiota follows a conserved program whereby the colonizing microbiome reflects the maternal vaginal microbiome, and become more similar to the maternal gut microbiome as offspring mature. Communities clustered using PCoA of the unweighted UniFrac distance matrix. The percentage of variation explained by the PC is indicated on the axes. Each point corresponds to a single community sample from a maternal body site or offspring colon, and color-coded by the indicated metadata. PCoA, principle coordinates analysis; PC, principle coordinates. N = 4–8 dams/site/day/treatment; N = 5–12 offspring/sex/day/treatment. (**b**) Average (±sem) unweighted UniFrac distance for pairwise comparisons between maternal gut and vaginal microbial communities at PN2. N = 4–8 dams/site/day/treatment; N = 5–12 offspring/sex/day/treatment. **p < 0.01. (**c**) Heatmap of mean relative abundances of 24-development discriminatory taxa identified by Random Forests. Cluster I corresponds to a microbial signature associated with early colonizers while Cluster II corresponds to a weaning microbial signature. Hierarchical clustering performed using the Spearman rank correlation distance matrix. See *Methods* for more detail on the Random Forests approach. (**d**) Random Forests identified development-discriminatory taxa are altered of EPS exposure. Differentially abundant taxa in colon samples from male and female control and EPS offspring at PN2, PN6, and PN28. See Supplemental Fig. 5B for additional comparisons. Barplots indicate median ± IQR. FCON, female control; MCON, male control; FEPS, female EPS; MEPS, male EPS; EPS, early prenatal stress; PN, postnatal day; IQR, Interquartile range. N = 5–12 offspring/sex/day/treatment; Mann Whitney U, *p < *0.05*, **p < *0.001*, ***p < *0.001*. PC, principal coordinate; PCoA, principal coordinates analysis. PN, postnatal day; M, male; F, female. FCON, female control; MCON, male control; FEPS, female EPS; MEPS, male EPS; EPS, early prenatal stress; PN, postnatal day. N = 5–12 offspring/sex/day/treatment.

As intestinal microbiota acquisition begins at birth through exposure of the maternal microbiota, we next determined whether these primary colonizers shared similarities with bacterial communities within the maternal gut, vagina, or a combination. Unweighted UniFrac matrices were calculated to determine distances between offspring gut microbiota and communities within the maternal gut and vagina, and then visualized using PCoA. Consistent with previous reports in humans, maternal vaginal bacterial profiles showed the greatest overlap with neonate gut profiles, while maternal gut bacterial profiles showed the greatest overlap with gut profiles of offspring at weaning (Fig. 3a)^33^,^35^,^37^. Additional unweighted UniFrac distance comparisons revealed that the community structure in the colon of control offspring at PN2 was more similar to PN2 control maternal vaginal samples than PN2 EPS maternal vaginal samples (*p* = 0.0027, permutation test; Fig. 3b). Conversely, colonic community structure in PN2 EPS offspring was more similar to PN2 EPS maternal vaginal samples compared with PN2 control maternal vaginal samples (*p* = 0.0046, permutation test; Fig. 3b).

### Effects of EPS on bacterial communities are sex-specific in offspring

Assembly of bacterial communities in the postnatal gut has been shown to parallel chronological age, and disruption in this synchrony has been associated with metabolic dysfunction^49^,^53^. As key features of the mouse model of early prenatal stress is male-specific metabolic dysfunction, we next determined assembly of bacterial communities is sexually dimorphic and altered by early prenatal stress. Colonic samples were obtained from control and EPS male and female pups at birth, during active breastfeeding, and weaning. Permutational multivariate analysis of variance using UniFrac distance measurements revealed a significant main effect of age (PERMANOVA pseudo-F = 17.61, *p* = 0.001), EPS by age interaction (PERMANOVA pseudo-F = 8.40, *p* = 0.001), and EPS by sex by age interaction (PERMANOVA pseudo-F = 4.33, *p* = 0.001). Post-hoc analysis revealed significant differences in the community structure of EPS males at PN28 compared with control males and EPS females at the same age (*p*s < 0.01, Supplemental Fig. 4B). Community diversity increased as a function of age in all offspring regardless of age and treatment across multiple measures of alpha diversity (Supplemental Fig. 4C).

Given the significant interaction between sex and chronological age on community structure, we next determined whether the assembly of bacterial communities differed between male and female offspring. To account for potential sex differences, two separate Random Forests models were regressed against chronological age. Constructing these age-discriminatory models for male and female offspring revealed that while many of the age-discriminatory taxa were shared between male and female offspring, their ranked importance in driving sex-specific maturation of the colonic bacteria differed (Supplemental Fig. 5A, Supplemental Table 2). Using this approach, we identified 27 age-discriminatory taxa that formed two distinct clusters: cluster 1 resembled an early life microbial community consisting of nine taxa, while cluster 2 resembled a weaning microbial community consisting of the remaining eighteen taxa (Fig. 3c,d). Further, eight age-discriminatory taxa exhibited significant sex differences (Supplemental Fig. 5B).

Using the microbial signatures identified from Random Forests analysis, we determined the sex-specific EPS effects on postnatal colonic microbiota maturation. Early colonizers were comprised of taxa within the Proteobacteria and Firmicutes phyla. At the genus level, *Aggregatibacter, Paenibacillus*, and *Lactobacillus* abundances were similar among male and female offspring, while sex differences in the relative abundance of *Streptococcus, Haemophilus*, and *Sphingobium* were observed (*p*s < 0.05, Mann Whitney U) (Supplemental Fig. 5B). EPS exposure disrupted the composition of the early colonizers *Lactobacillus* and *Streptococcus* independent of sex (*p*s < 0.05, Mann Whitney U) (Fig. 3d). These early colonizers were rapidly replaced by exclusive dominance of *Lactobacillus* and *Aggregatibacter*, regardless of sex and treatment (Fig. 3d, Supplemental Fig. 5B). By weaning, the abovementioned expansion in microbial diversity and richness during this period was related to the replacement of facultative anaerobes such as *Aggregatibacter, Lactobacillus*, and *Streptococcus* by a vast community of strictly obligate anaerobes (Fig. 3d, Supplemental Fig. 5B). Interestingly, sex differences also reemerged at weaning (e.g., PN28). *Mucispirillum, Odoribacter*, and *Desulfovibrio* were enriched in females at weaning, while *Dehalobacterium* and *Flexispira* were enriched at weaning in males (Fig. 3d, Supplemental Fig. 5B). Early prenatal stress exposure disrupted sex differences in *Odoribacter, Desulfovibrio, Flexispira*, and *Mucispirillum* abundance, while *Lachnospiraceae* and *Clostridiales* abundance were increased only in EPS males relative to control males (Fig. 3d, Supplemental Fig. 5B). In addition to the EPS sex-specific effects on the gut microbiota composition at weaning, a main effect of EPS on the relative abundance of the Bacteroidetes *Rikenella* was also observed (*p* < 0.001) (Fig. 3d, Supplemental Fig. 5B). Collectively, our results demonstrate that maternal stress during the first week of pregnancy shapes sex-specific maturation of the colonic microbiota.

To relate composition shifts to functional capacity, PICRUSt analysis was used to predict KEGG functional categories, and similar to the approach above, separate Random Forests regressions were constructed to identify functional shifts associated with maturation (Supplemental Fig. 6A). Thirty-four age-discriminatory pathways were identified (Supplemental Fig. 6A). Early colonizers were enriched in functional pathways related to metabolism and degradation of amino acids, ketone bodies, and the short chain fatty acids butyrate and propionate (Supplemental Fig. 6B). Despite the limited representation of microbial communities during the lactation (i.e., PN 6), numerous unique microbial metabolic pathways were enriched only during this period, including primary bile acid biosynthesis, ascorbate and aldarate metabolism, alpha-Linolenic metabolism, tryptophan metabolism, steroid hormone biosynthesis, and lysine degradation (Supplemental Fig. 6B). Consistent with the expansion of community diversity as a function of chronological age, bacterial functional capacity was characterized at weaning by extensive enrichment of carbohydrate and amino acid metabolic pathways (Supplemental Fig. 6B).

## Discussion

Early life host-microbe interactions disproportionally contribute to later health outcomes including susceptibility and resistance to infection, predisposition to inflammatory and metabolic disorders, and changes to neural circuits that control stress and affect, yet remarkably little is known about the maternal factors that may influence this process^19^,^54^,^55^,^56^,^57^,^58^,^59^,^60^. Although maternal stress experience during pregnancy is associated with profound dysregulation of pathways along the gut-brain axis, far less is known regarding the direct impact of stress on maternal bacterial communities that, in turn, shape these health outcomes in offspring^15^,^16^,^17^,^18^,^30^,^61^,^62^,^63^,^64^,^65^. Thus, alterations to temporal and spatial dynamics of maternal gut and vaginal bacterial communities during pregnancy and postpartum may represent a novel mode of stress reprogramming of offspring outcomes.

To determine whether early gestation stress exhibits a lasting impact on maternal fecal bacterial communities across pregnancy, fecal samples were collected at daily intervals from the initiation of pregnancy to term. Time-series profiling revealed that the murine maternal fecal bacteria undergo remodeling over the course of pregnancy. Surprisingly, two distinct windows demarcate community structure, composition, and functional capacity during pregnancy that parallel distinct metabolic adaptations, such as weight gain, that occur during pregnancy^20^. One question raised by our observations relates to host factors that may contribute to maternal gut bacterial community temporal patterns. Complex hormonal, endocrine, and immune interactions in the pregnant female emerge between gestation day 8 and 11, suggesting factors specific to pregnancy may mediate community dynamics. Feto-placental vascularization occurs during this window, a process during which the placenta begins to invade the uterus, gains contact with maternal circulation, and substrate transport from maternal circulation to the fetus is initiated^66^. A possible explanation is that maternal and fetal signals converge upon certain groups of bacteria that provide a pool of substrates important for fetal growth and development^23^. In this regard, selection and subsequent stabilization of these microbial communities may represent an adaptation to ensure sequestration of substrates important for offspring development. Thus, the capacity of such signals to impose selective pressures upon maternal microbial composition and function during pregnancy is intriguing and warrants future study.

Our observations of lasting stress alterations to maternal gut bacterial diversity and composition reveal a novel mode of prenatal stress reprogramming. Bacterial diversity and composition change abruptly in response to stress, which may be related to previous observations that chronic stress exaggerates the normal physiological immunosuppression and metabolic syndrome that occurs during pregnancy^67^. Thus, stress-related changes to the nutritional, endocrine, or immune environment of the maternal gut might explain the simultaneous bloom of facultative anaerobes *Mucispirillum* (phylum Deferribacteres) and *Desulfovibrionaceae* (phylum Proteobacteria) at the expense of some obligatory anaerobes^31^,^68^,^69^. The combined capacity to degrade mucin (*Mucispirillum*) and production of hydrogen sulfide (*Desulfovibrionaceae*) may provide a putative link between stress during pregnancy and intestinal inflammation^70^,^71^,^72^. Taken together, these results suggest that stress during the first week of pregnancy induced a coordinated shift in the maternal fecal bacteria community structure, particularly during periods of pregnancy when community structure is most variable.

Dynamic shifts in the functional capacity of fecal bacterial communities parallel well-established metabolic adaptations in pregnancy, implicating pregnancy as an important period of host-microbe synchrony^20^. Accumulation of nutrients and lipid stores occurs during the early phase of pregnancy when offspring growth is limited^20^. Similarly, we observe microbial pathways involved in the biosynthesis of amino acids and lipids enriched during the early phase of pregnancy. To support the demand underlying the exponential growth phase of fetal development during the latter portion of pregnancy, maternal metabolic processes shift to a catabolic state that is characterized by accelerated lipolysis, gluconeogenesis, and ketogenesis^20^. The enrichment of predicted functional pathways involved in gluconeogenesis and metabolism of fatty acids during the late phase of pregnancy may highlight an important role of bacterial communities in modulating the metabolic demand of fetal growth and development. Enrichment of pathways involved in amino acid and glycerophospholipid metabolism observed during the early pregnancy window was disrupted by maternal stress experience. Similarly, upregulation of lysine biosynthesis pathways during late gestation was disrupted in females exposed to stress. In contrast, stress increased the abundance of pathways involved in biosynthesis of fatty acids and degradation of branched chain amino acids, suggesting the possibility that stress during pregnancy may induce an alternate metabolic state of the bacterial communities, that, in turn, may impact availability of metabolites. Building on these community-level analyses, additional mechanistic studies are now needed to identify stress-altered bacterial strains, the metabolites produced by these strains, and their importance in normal fetal growth and development.

In addition to maternal gut communities, stress may also alter vaginal bacterial communities that provide the primary inoculum during birth. To determine the lasting effect of maternal stress experience on vaginal bacterial communities, vaginal fluid samples were collected prior to mating, at day 7 of pregnancy, and 24 h postpartum. Surprisingly, *Aggregatibacter* of the Pasteurellaceae family within the phylum Proteobacteria dominated the communities of the mouse vagina. Although communities in the human vagina are dominated by the acidophilic lactobacilli, this appears to be largely impacted by vaginal pH: as vaginal pH shifts towards neutral, the communities reflect higher diversity and loss of *Lactobacillus* dominance without consequences on vaginal health^73^. Further, lactobacilli dominance does not appear to reflect the vaginal ecosystem of non-human primates and livestock, thus, it is not surprising that mouse vaginal communities were not dominated by *Lactobacillus*^74^,^75^. Nevertheless, the vaginal microbial composition shifted considerably during the first week of pregnancy, from near complete dominance of *Aggregatibacter* in nonpregnant females to a distinct community state type composed of *Aggregatibacter, Prevotella, Clostridiales*, and *Lachnospiraceae*. Dominance of *Aggregatibacter* reemerged in the early postpartum period, collectively highlighting temporal dynamics of the mouse vaginal microbiota during pregnancy. Building upon our previous work, we now show that stress during pregnancy alters bacterial community dynamics of the maternal vagina during pregnancy and the postpartum period^18^. Moreover, consistent with previous reports in rodents and humans, significant overlap was observed between the maternal vaginal and neonate colonic microbiota^32^,^33^,^34^,^35^,^76^. Similarities between mother-offspring microbiota were dependent on maternal body site and age of offspring, whereby neonate colonic microbiota resemble the maternal vaginal microbiota immediately following birth and become more similar to the maternal gut microbiota as a function of age^33^,^34^,^35^.

To determine whether maternal stress experience shapes assembly of offspring colonic microbiota, community composition was evaluated in offspring colon samples at birth, during active breastfeeding, and at weaning. Microbial diversity increased as a function of age and independent of sex or treatment. Conversely, the structure of bacterial communities appears to be mediated through a complex interaction between age, sex, and treatment. EPS exposure significantly altered community structure in males at weaning compared with control males, and this alteration parallels the emergence of key EPS phenotypes such as changes in metabolism, stress responsivity, and cognition^25^,^26^,^27^,^45^,^46^,^47^,^48^.

To further examine the lasting impact of maternal stress exposure on offspring microbial communities, we used Random Forests models to identify sex- and age-discriminatory taxa that define a normal program of microbial assembly. This approach showed that postnatal maturation in control mice followed a patterned progression, characterized by colonization of γ-Proteobacteria and *Lactobacillus* immediately after birth that is rapidly replaced by *Lactobacillus* during exclusive breastfeeding, and an adult-like microbiota composition emerged following transition to solid chow^35^,^49^,^76^. Transient γ-Proteobacteria colonization at birth is essential in the regulation of granulocytosis, neutrophil homeostasis, and resistance to neonate sepsis^39^. Subsequent induction of Proteobacteria-specific immunoglobulin A appears to drive the rapid suppression of this phylum and primes the intestinal niche for replacement by Firmicutes such as *Lactobacillus*^76^,^77^. The transition at weaning likely involves complex interactions between microbial adaptations to dietary and nutritional shifts, immune maturation, and host transcriptional programming^78^. Sex differences in the proportional representation of age-discriminatory taxa may suggest that offspring sex is an important host factor in modulating microbial assembly and maturation of processes mediating host-microbe interactions^79^,^80^. Interestingly, taxa observed at higher abundance in weanling females – *Mucispirillum, Desulfovibrio*, and *Odoribacter* – have been shown to be part of a colitogenic gut microbiome^81^. Whether this baseline sex difference reflects heightened susceptibility, immunological tolerance, or metabolic status in females warrants further study^81^,^82^,^83^,^84^,^85^. Further, early prenatal stress exposure disrupted sex differences in *Mucispirillum, Desulfovibrio*, and *Odoribacter*, with exposed males exhibiting a female-typical pattern. The observed disruption of established sex differences by dysmasculinizing male offspring is a hallmark of our mouse model of early prenatal stress, including sex-specific reprogramming of hypothalamic and limbic circuits and dysregulation of stress responsivity, cognitive dysfunction, and post-pubertal growth^26^,^45^,^48^. Taken together, these data suggest that disruption of sex differences in the gut microbiome precedes the emergence of key EPS phenotypes in male offspring, implicating the gut microbiome as a putative and early predictor of offspring dysfunction in adulthood.

As with any descriptive dataset, there are limitations here in making causal inferences. The current data has a reliance on data generated from 16S rRNA maker gene sequencing and predictive algorithms to drive conclusions regarding the impact of the maternal and offspring bacterial communities as a novel contributor to the EPS phenotype. Additional work that combines time-series profiling with bacterial strain-level resolution sequencing may identify temporal and spatial dynamics of bacterial strains and functional categories by stress in dam and offspring. Additionally, the current study does not provide functional links between these alterations in bacterial community composition and metabolite availability in dam and offspring. While previous work in our laboratory has demonstrated significant shifts in metabolite availability in EPS offspring at postnatal day 2, additional work is required to identify whether stress-mediated alterations to maternal and fetal metabolites during the prenatal window represent a causal link to key aspects of the EPS phenotype^18^.

With these important caveats in mind, our data provide novel insight into the role of chronic stress on prenatal and postnatal development via the maternal gut and vaginal microbiome, respectively. Stress during the first week of pregnancy induces rapid and lasting alterations to the microbial landscape within the maternal gut and vagina. Further, maternal stress experience impacts assembly of the postnatal colonic microbiome in a sex-specific manner and these alterations precede the emergence of key EPS phenotypes, such as stress pathway dysregulation, cognitive deficits, and metabolic changes in these males as adults. Collectively, our results suggest stress reprogramming of offspring development may occur via the maternal microbiota through two novel mechanisms. First, stress alterations to maternal gut microbiota composition and function during pregnancy may impact the available pool of microbe derived metabolites and substrates that are necessary for normal prenatal growth and development^86^. Second, transmission of stress-altered vaginal microbiota may disrupt neonate-microbe interactions necessary for normal immune education, metabolism, and neurodevelopment^37^,^41^,^42^,^43^,^44^. Gut colonization by these stress-altered vaginal microbiota may, in turn, prime the intestinal niche for successors that are associated with greater disease risk later in life, particularly those disorders that exhibit a strong sex bias in onset, severity, and treatment outcomes. Our results open possibilities for future studies on defining mechanisms of these associations, and producing studies in which causality can be demonstrated between the neonate microbiome and the developing brain.

## Methods

### Experimental animals

Mice used in these studies were on a mixed C57BL/6:129 background that are maintained in-house as an outbred mixed colony. A total of 30 mixed colony breeding pairs were used for these studies, and females were checked daily at 700 EST for copulation plugs. Dams were housed under a 12 h light/day photoperiod with a standard chow diet (Purina Rodent Chow, St. Louis, MO; 28.1% protein, 59.8% carbohydrate, 12.1% fat) and *ad libitum* access to water. Noon on the day that the plug was observed was considered to be embryonic day 0.5 (E0.5). Samples from one male and one female per litter were used for all subsequent analyses. All experiments were approved by the University of Pennsylvania Institutional Animal Care and Use Committee and performed in accordance with *National Institutes of Health Animal Care and Use Guidelines*.

### Early prenatal stress

Administration of chronic variable stress was performed as previously described^25^,^26^,^27^,^45^,^46^,^47^,^48^. Dams were randomly assigned to treatment groups to receive stress during days 1–7 of gestation (EPS; n = 8) or to a control (n = 5) unstressed group. Following confirmation of a copulation plug, pregnant mice assigned to the EPS group experienced each of the following stressors on different days: 60 min of fox odor exposure (1:5,000 2,4,5-trimethylthiazole; Acros Organics), 15 min of restraint (beginning at 1300 EST) in a modified 50-mL conical tube, 36 h of constant light, novel noise (White Noise/Nature Sound-Sleep Machine; Brookstone) overnight, three cage changes throughout the light cycle, saturated bedding (700 mL, 23 °C water) overnight, and novel object (eight marbles of similar shape and color) exposure overnight. Stressors were selected to be non-habituating and did not induce pain or directly influence maternal food intake, weight gain, behavior, litter size, or sex ratio^25^,^26^,^27^,^45^,^46^,^47^,^48^.

### Sampling strategy

The fecal, vaginal, and colonic high-resolution sampling schedule is shown in Fig. 1a. Following confirmation of the mating plug, females were randomly assigned to either control or stress (EPS) groups and daily fecal samples were collected at the same time each day to control for diurnal confounds. A vaginal fluid sample was collected following cessation of the chronic variable stress protocol (i.e., gestation day 7.5). Twenty-four hours after birth, an additional vaginal fluid sample was collected to assess similarity between maternal microbiota and pup gut microbiota profiles. On postnatal day (PN) 2, 6 and 28 colon samples from 1 male and 1 female offspring per litter were collected for sequencing. For these experiments in offspring, bacterial enumeration by 16S rRNA sequencing was conducted from colonic scrapes that include the loose colonic contents and mucus. These time points were selected to assess neonate microbial structure and function during key periods of assembly: colonization (PN2), breastfeeding (PN2), and weaning (PN28). Maternal fecal (n = 4–8 females per treatment sampled every day, yielding a total of 228 unique fecal samples), maternal vaginal (n = 5–8 /treatment/time point; 39 unique vaginal fluid samples in total), and offspring distal colon samples (n = 5–8 offspring/sex/treatment/time point, for a total of 80 colon samples) comprise the total number of samples used in this study. To control for potential confounds of the housing and the cage environment in the assessment of EPS effects on the microbiota, several precautions were taken: first, in the bacterial community composition assessment in maternal fecal and vaginal samples, females were singly housed during the entire duration of the pregnancy; and, second, in the bacterial communities assessment within offspring colonic samples, one male and one female offspring were collected at each time point from a different dam, such that the sample size correspond to cage number.

### DNA isolation, amplification, and sequencing of the 16S rRNA gene

Fecal, vaginal fluid, and colonic samples were rapidly frozen in liquid nitrogen and subsequently stored at −80 °C before DNA extraction. Genomic DNA from fecal, vaginal and colon samples was isolated using the Stratec PSP Spin Stool DNA Plus kit using the difficult to lyse bacteria protocol from the manufacturer (STRATEC Molecular GmbH, Berlin, Germany). For each set of extractions, one blank swab exposed to laboratory air was processed as a negative control. The V4 region of the 16S rRNA gene was amplified using barcoded primers for the Illumina platform as previously described^87^. Sequencing was performed on a MiSeq instrument (Illumina, San Diego, CA) using 250 base paired-end chemistry at the University of Pennsylvania Next Generation Sequencing Core. Mock communities, consisting of genomic DNA from 12 ATCC strains, were included as a positive control and for run-to-run quality control. Quality filtering and chimera checking yielded 15,471,769 quality-filtered sequences with a mean ± SD depth of 24,326 ± 4,1875 sequences per sample.

### 16S rRNA gene analysis

Paired-end reads were assembled and quality filtered to include sequences with a *Q* score ≥30. mothur v. 1.36.1 was employed to remove sequences <248 bp and >255 bp in length and sequences with homopolymers >10 bp in length^88^. QIIME v. 1.8 was used for further downstream processing and analyses^89^. OTUs were defined using 97% sequence similarity with CD-HIT, and a representative sequence from each OTU containing ≥10 sequences was chosen for downstream analyses (based on the most abundant sequence). Chimeric sequences were removed using ChimeraSlayer. Representative sequences were assigned to genera using the Ribosomal Database Project (RDP) classifier v 2.2, multiple sequence alignment was performed using PyNAST, and a phylogeny was built with FastTree. The samples were rarified to 1,000 sequences per sample for calculating alpha- and beta-diversity metrics.

### Bioinformatics quality assurance

For quality control purposes, water and processed blank samples were sequenced and analyzed through the bioinformatics pipeline. Taxa identified as cyanobacteria or ‘unclassified’ to the phylum level were removed. For further quality control assurance and to ensure run-to-run reproducibility, genomic DNA from abovementioned mock communities were sequenced and the expected sequences were compared to the obtained sequences.

### Identifying microbial communities involved with pregnancy and offspring postnatal maturation using Random Forests

Random Forests, a machine-based-learning algorithm, was used to regress microbiota relative abundance in the time-series profiling of mothers and offspring against day of pregnancy or chronologic age, respectively. The R package ‘randomForest’, ntree = 1000 was used with a default mtry parameter of p/3 where p is the number of input 97%-identity OTUs (features)) as previously described^49^. This approach allows for the identification of taxa that discriminate between different periods of interest, including stages of pregnancy and age of development in offspring. OTU importance was ranked by the percent decrease in prediction accuracy of the model that occurs when that OTU is removed. To estimate the minimal number of top ranking discriminatory taxa required for prediction, fivefold cross-validation using the “rfcv” function implemented in the ‘randomForest’ package was applied over 100 iterations^49^. A rarefied OTU table at 1,000 sequences per sample served as input data. The model derived from control maternal and offspring microbiota was then applied to microbiota from stress-exposed mothers and male and female offspring. The R package “Heatplus” was used to plot OTU relative abundances as heatmaps.

### Predicted metagenomic analysis based on 16s rRNA sequencing data

The predicted functional capacity of the maternal gut and vaginal microbiota, as well offspring gut microbiota, was predicted using phylogenetic investigation of communities by reconstruction of unobserved states (PICRUSt)^52^. This algorithm extended ancestral-state reconstruction algorithm to predict which gene families are present and then combines gene families to estimate the composite metagenome. The analysis includes the following steps. (i) Obtaining a closed reference OTU table; (ii) Normalization of the OTU table; (ii) Predicting the functions for metagenomes. The last step produces the actual metagenome functional predictions (KEGG Orthologs) for a given OTU table, which multiplies each normalized OTU abundance by each predicted functional trait abundance to produce a table of functions by samples. As our *a priori* hypotheses are directly related to the metabolic contribution of the microbiota to the host, the predicted metagenome dataset was filtered to only include KEGG Orthologs that mapped on to the “Metabolism” category. Random Forests were applied to this filtered metagenome dataset to identify functional categories that shift as a function of stage of pregnancy or postnatal age, and then impact of stress on the proportional representation of these predicted microbial metabolic pathways was assessed.

### Additional statistical methods

Alpha diversity and beta diversity are reported as mean ± SEM. Taxonomic relative abundances are reported as the median ± interquartile range (IQR). Permutational multivariate analysis of variance using distance matrices (PERMANOVA) was used to analyzed effects of age, sex, treatment, and interactions on unweighted UniFrac distances^90^. Linear regression models were used to predict the best fit for community diversity as a function of day of pregnancy. Repeated measures were used to assess significant changes in alpha diversity across gestation, followed by pairwise comparisons adjusted for multiple comparisons. For pairwise comparisons, *P* values were calculated using the Kruskal-Wallis test, Wilcoxon rank-sum test, or Mann Whitney U in GraphPad.

## Additional Information

**How to cite this article**: Jašarević, E. *et al*. Stress during pregnancy alters temporal and spatial dynamics of the maternal and offspring microbiome in a sex-specific manner. *Sci. Rep.* **7**, 44182; doi: 10.1038/srep44182 (2017).

**Publisher's note:** Springer Nature remains neutral with regard to jurisdictional claims in published maps and institutional affiliations.

## Supplementary Material

Supplementary Information

Supplemental Table 1
