# Supplementary material for: Stress during pregnancy alters temporal and spatial dynamics of the maternal and offspring microbiome in a sex-specific manner
Source: Sci Rep. 2017 Mar 7;7:44182. doi: 10.1038/srep44182 (PMC5339804; doi:10.1038/srep44182)

Stress during pregnancy alters temporal and spatial dynamics of the maternal and offspring microbiome in a sex-specific manner

Eldin Jašarević[1,2], Christopher D. Howard[1,2], Ana M. Misic[2,3], Daniel P. Beiting[2,3], and Tracy L. Bale[1,2*]

[1]Department of Biomedical Sciences, [2]Center for Host-Microbial Interactions, [3]Department of Pathobiology, School of Veterinary Medicine, University of Pennsylvania, Philadelphia, PA 19104

Corresponding author
*tbale@vet.upenn.edu

Supplemental Figure 1. Metabolic adaptations in pregnancy are correlated with bacterial community dynamics. (a) High-resolution sampling strategy to interrogate the lasting impact of stress during pregnancy on maternal and offspring microbial communities. **(b)** Shannon diversity is significantly correlated with maternal body weight gain across pregnancy, $r^2 = 0.11$, $p = 0.011$. N = 4 – 8 females sampled daily per treatment.

Supplemental Figure 2. Temporal effects of stress on maternal fecal microbial composition during pregnancy. (a) Inferring the impact of early pregnancy stress on the maternal fecal bacterial communities. Thirteen taxa identified by applying Random Forests to fecal sample relative abundance data against stage of pregnancy in control females only. Shown are 97%-identity OTUs with their deepest level of taxonomic annotation for the 16S V4 region (i.e., genus), ranked in descending order of their importance to the accuracy of the model. The insert shows fivefold cross-validation error as a function of the number of 97%-identity OTU's to identify the lowest number of taxa significantly contributing to the pregnancy discriminatory model. **(b)** Validation of stress effects on the proportional representation of taxa that discriminate between early and late pregnancy. Differentially abundant taxa in fecal samples from control and stress-exposed females during early and late pregnancy. Barplots indicate median ± IQR. CTL, Control; EPS, early prenatal stress; N = 4 – 8/day/treatment, 228 samples in total; Nonparametric *t*-Test, * $p < 0.05$, ** $p < 0.001$, *** $p < 0.001$. **(c)** Functional capacity of gut bacterial communities are altered by maternal stress during. Correlation matrices between PICRUSt-generated functional pathways and genus level bacterial abundance were calculated and plotted subject to maternal stress exposure. Only genera and functional pathways identified by Random Forest that differ between control and stress-exposed females are depicted. The upper part of the graph represents control dams, while the lower part represents stress exposed females. Empty boxes indicate non-significant correlations, while filled boxes are indicative of a significant correlation, with a *P*-value≤0.05 by Kendall's test. The shading intensity of the bubble, along with size, is indicative of the Kendall rank correlation coefficient between matrices. Blue designates a negative correlation while red designates a positive correlation. CTL, Control; EPS, early prenatal stress; N = 4 – 8/day/treatment, 228 samples in total.

Supplemental Figure 3. The gut microbiome exhibits distinct functional capacity during pregnancy that is disrupted by stress experience. **(a)** Inferring the impact of chronic variable stress on the maternal gut microbiota functional capacity during pregnancy. Using 97%-identity closed reference OTU's rarefied at 1000 sequences per sample as input data, predictive functional profiling was conducted using Phylogenetic Investigation of Communities by Reconstruction of Unobserved States (PICRUSt). The resulting data was filtered only for metabolism related pathways. To identify predictive functional pathways that discriminate stage of pregnancy, Random Forests were then applied to predictive metagenome relative abundance against the stage of pregnancy in control females only. Shown are the predictive microbial metabolic pathways ranked in descending order of their importance to the accuracy of the model. The insert shows the fivefold cross-validation error as a function of the number of predicted microbial metabolic pathways to identify the lowest number of taxa significantly contributing to the pregnancy discriminatory model. **(b)** A heatmap of mean relative abundance of the pregnancy-stage discriminating microbial metabolic pathways plotted against stage of pregnancy of control and stress-exposed females. Hierarchical clustering performed using the Spearman rank correlation distance matrix. N = 4 – 8/day/treatment, 228 samples in total.

Supplemental Figure 4. Postnatal bacterial community dynamics in offspring. (a) Assembly of colonic microbiota mediated by chronological age. Communities clustered using PCoA of the unweighted UniFrac distance matrix. The percentage of variation explained by the PC is indicated on the axes. Each point corresponds to a single community sampled from the offspring colon, and color-coded by the indicated metadata. PERMANOVA p-values based on 999 Monte Carlo simulations. **(b)** Average (± sem) unweighted UniFrac distance for pairwise comparisons between offspring microbial communities at PN2, PN6, and PN28 N = 4 – 8 dams/site/day/treatment; N = 5 – 12 offspring/sex/day/treatment. Mann Whitney U, * $p < 0.05$, ** $p < 0.01$, *** $p < 0.001$. **(c)** Average (± sem) of alpha diversity measures - Shannon Diversity Index, Phylogenetic Diversity, Dominance, and McIntosh Evenness – contrasted by age, sex, and treatment demonstrating that community diversity increases significantly at weaning, independent of sex or EPS treatment. N = 5 – 12 offspring/sex/day/treatment.

Supplemental Figure 5. **Random Forest models identified taxa with sex differences in proportional representation during critical maturational periods. (a)** Inferring the impact of early prenatal stress on sex-specific postnatal development of the gut microbiota. Twenty-four development-specific taxa identified by applying Random Forests to colon sample relative abundance data against age in control males (left) and control females (right). Shown are 97%-identity OTUs with their deepest level of taxonomic annotation for the 16S V4 region (i.e., genus), ranked in descending order of their importance to the accuracy of the model. The insert shows fivefold cross-validation error as a function of the number of 97%-identity OTU's to identify the lowest number of taxa significantly contributing to the age discriminatory model. See *Methods* for detail on Random Forests modelling parameters. **(b)** Sex differences in development-discriminatory taxa are altered of EPS exposure. Differentially abundant taxa in colon samples from male and female control and EPS offspring at PN2, PN6, and PN28. Barplots indicate median ± IQR. FCON, female control; MCON, male control; FEPS, female EPS; MEPS, male EPS; EPS, early prenatal stress; PN, postnatal day. N = 5 – 12 offspring/sex/day/treatment; Mann Whitney U, * $p < 0.05$, ** $p < 0.001$, *** $p < 0.001$.

Supplemental Figure 6. Sex- and age- discriminatory functional pathways identified by Random Forest. (a) To test functional consequences of EPS, a similar Random Forests approach was used to first identify predicted KEGG metabolic pathways that shift in control male and female offspring. Shown are predictive microbial metabolic pathways ranked in descending order of their importance to the accuracy of the model in control female (left) and male (right) offspring. The insert shows the fivefold cross-validation error as a function of the number of predicted microbial metabolic pathways to identify the lowest number of taxa significantly contributing to the pregnancy discriminatory model. **(b)** A heatmap of the mean relative abundance of the sex-by age- discriminatory microbial metabolic pathways plotted against stage of pregnancy of control and stress-exposed females. Hierarchical clustering was performed using the Spearman rank correlation distance matrix. FCON, female control; MCON, male control; FEPS, female EPS; MEPS, male EPS; EPS, early prenatal stress; PN, postnatal day. N = 5 – 12 offspring/sex/day/treatment

Supplemental Figure 1

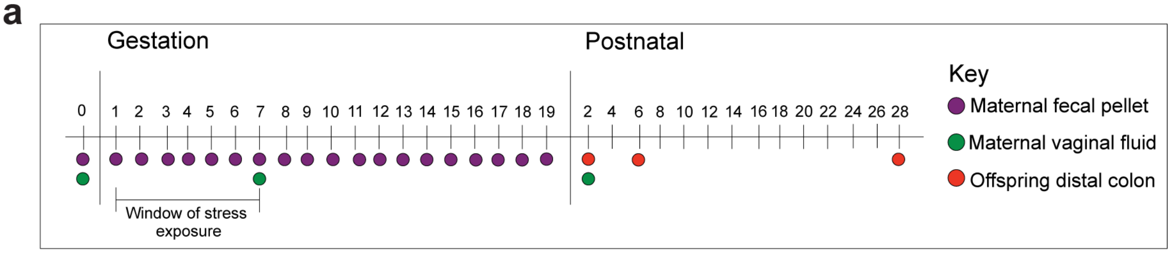

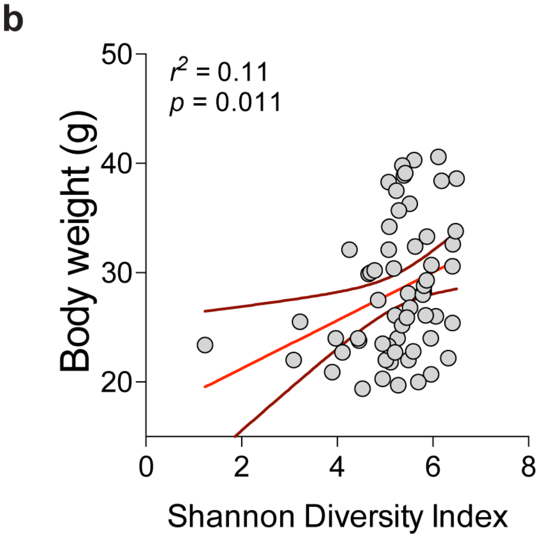

Supplemental Figure 2

a

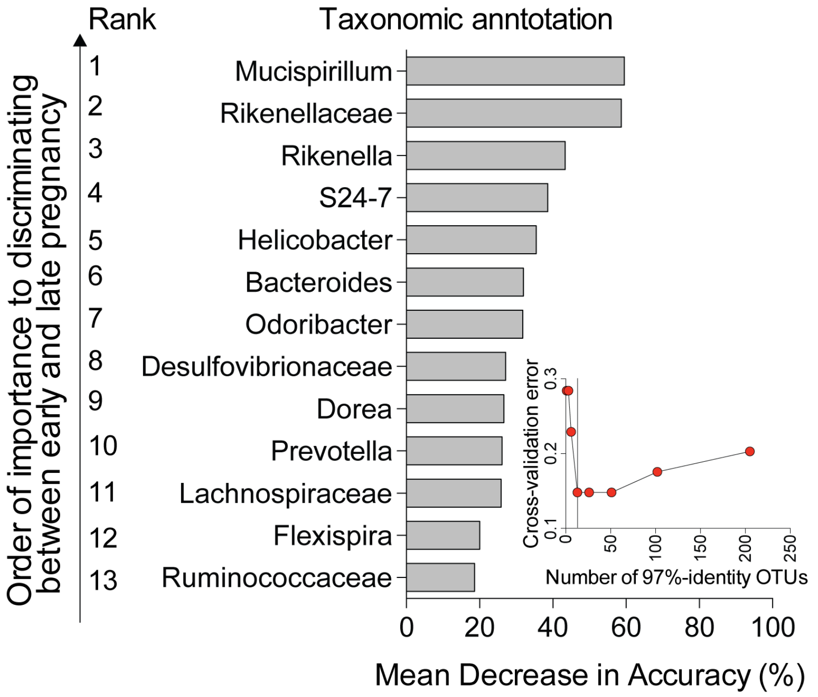

b

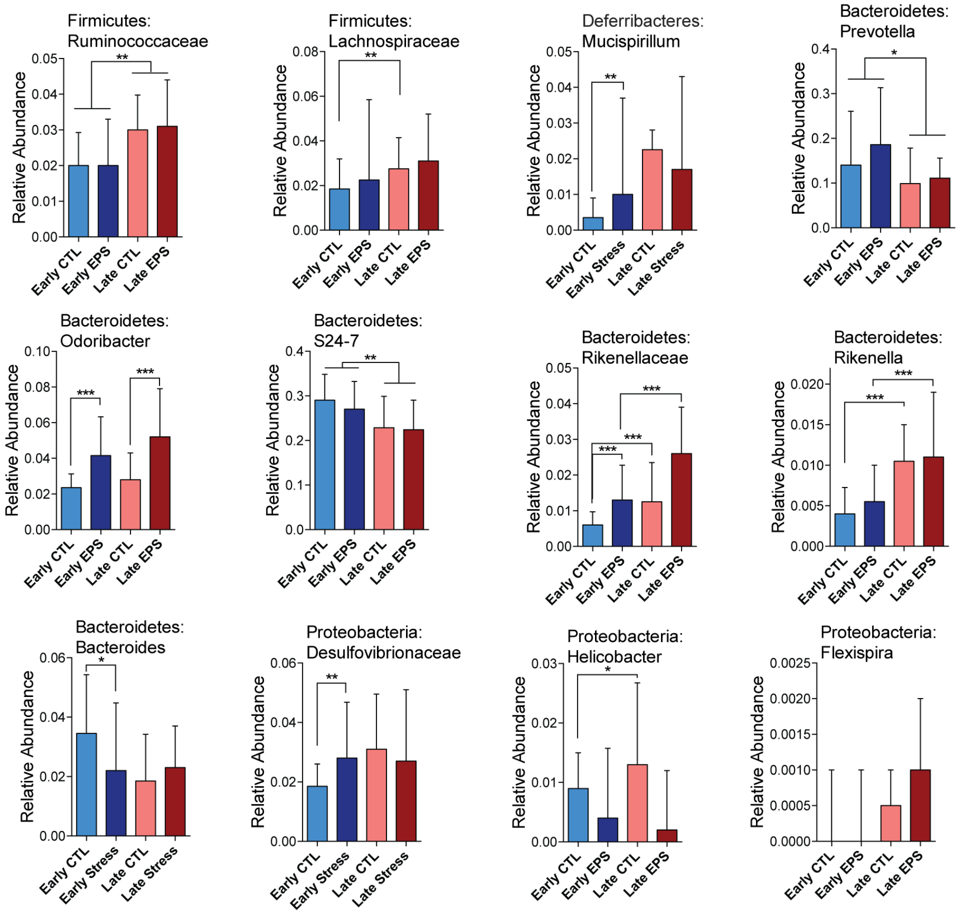

Supplemental Figure 3

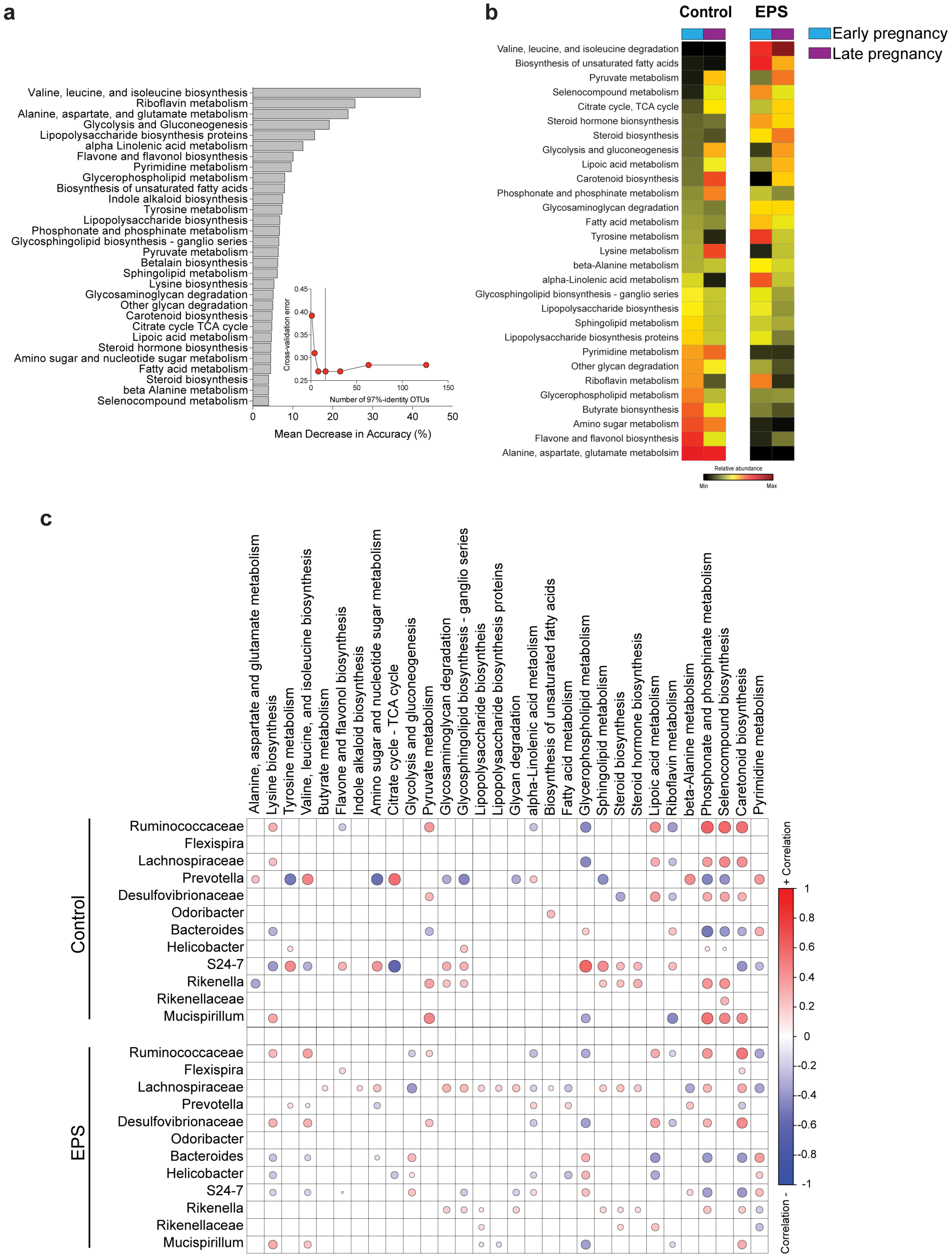

Supplemental Figure 4

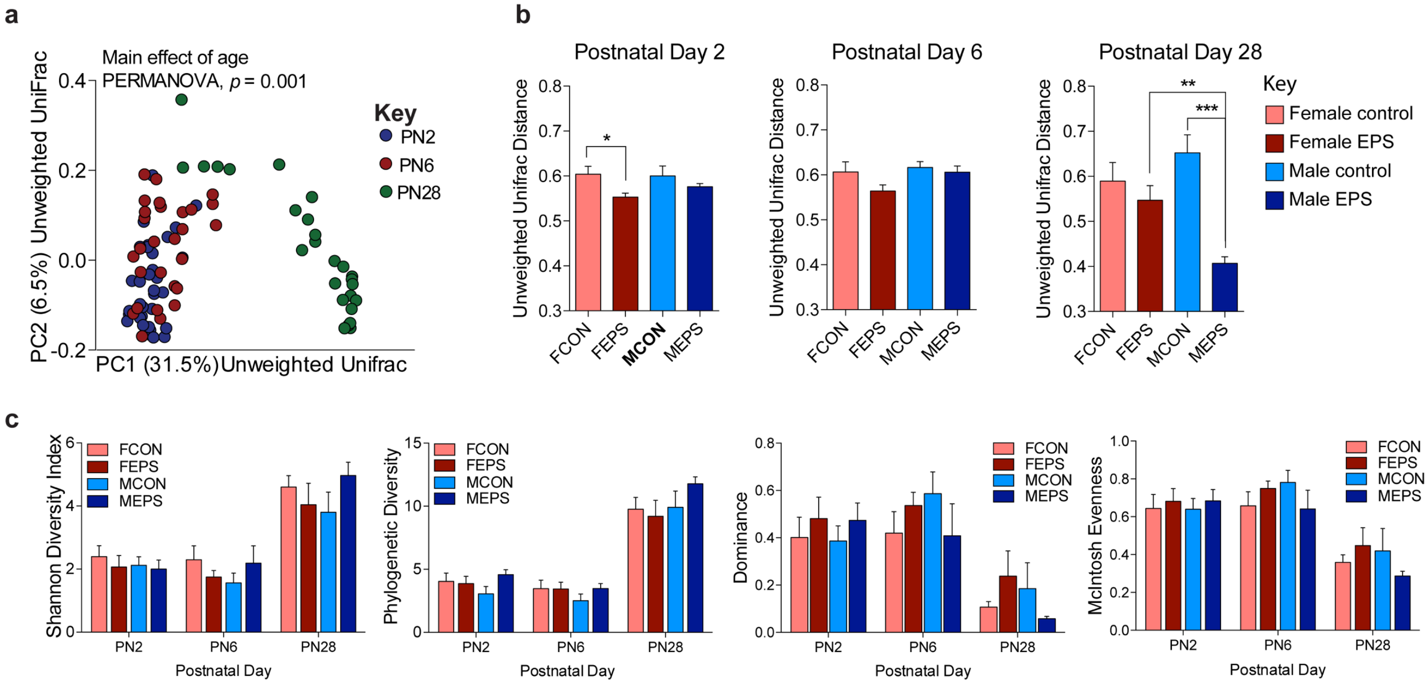

Supplemental Figure 5

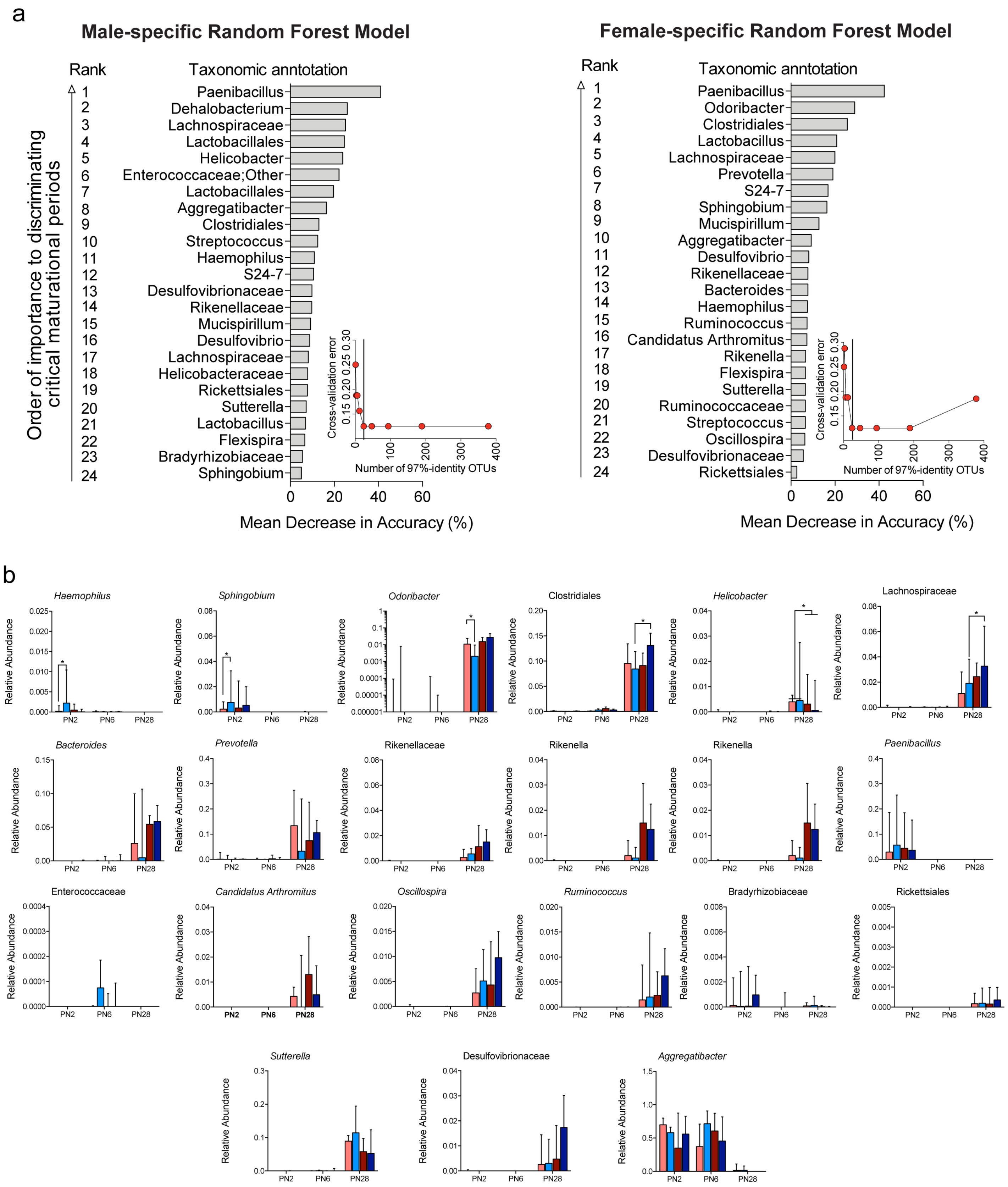

Supplemental Figure 6

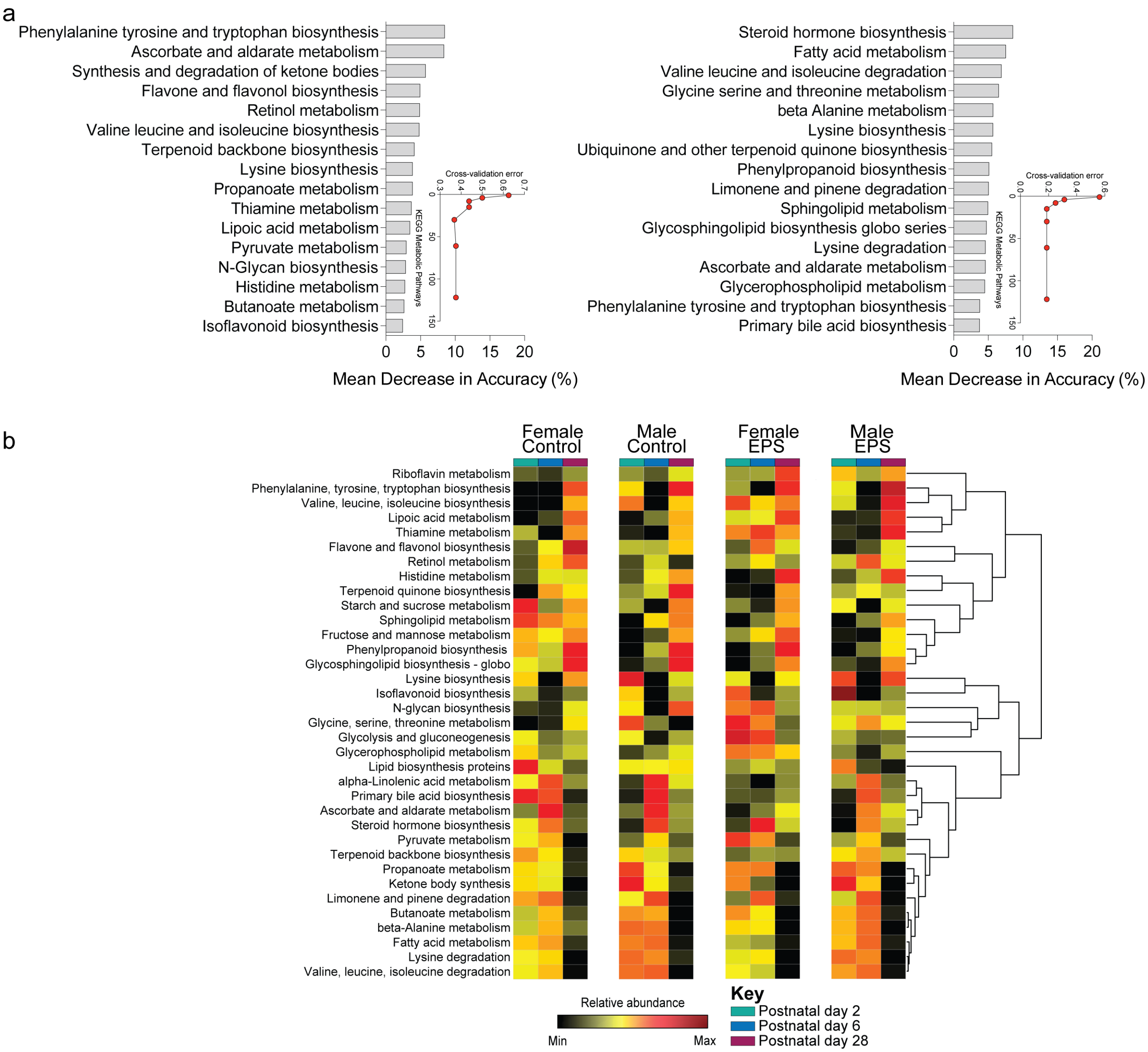

Supplement: Supplementary Information [file srep44182-s1.pdf]
